# Tait’s Operation in Texas

**Published:** 1886-11

**Authors:** 


### CORRESPONDENCE.

### TAIT'S OPERATION IN TEXAS.

**Note from Dr. Wilkinson, President of Galveston Club.**

EDITOR DANIEL'S MEDICAL JOURNAL.

DEAR SIR:

IN your October number you allude to a meeting of the Galveston Medical Club wherein you quote me as having stated in connection with a subject up before that body for discussion that "Four cases of Tait's operation had been performed in Texas," and you proceeded to show that several other similar operations had been performed etc., etc.

Now, in justice to myself and to the editor of the "Courier-Record of Medicine," who was only reporting what he had heard, I desire to state what was said, and my reasons for such statement.

In announcing to the Club that four cases of Tait's operation had been performed in Texas, it was not, by any means, intended that *only* four such cases had ever occurred in our entire State. On the contrary, the writer was of the opinion that others had been performed. But this he wished to emphasize, viz.: that *only the report of four such cases had come to his knowledge.* Up to the first of the present year, but seven cases of *oophorectomy* had been reported. (See Cupples' report on Surgery to State Association 1886. How many of these were Battey's, and how many were Tait's I cannot state; probably three of the former, and four of the latter; possibly fewer of Tait's. Be this as it may, certainly not more than four or five salpingo-oophorectomies had, up to August 1, of the present year, been reported in any Texas Medical Journal, if indeed in any other, though many more may have been performed.

It is a source of regret to us all that physicians do not report such cases, as the credit of Texas surgery could not fail to be enhanced by such publicity.

It was with this very object in view, to-wit: In order to bring out the experiences of other gentlemen in this department of Gynæcology, that the writer furnished the list of Tait's operations that had been brought to his particular attention.

I am induced to believe that few, if any, of Dr. Cupples' cases, or in fact, few of any other operative cases, have ever found light in either of our home journals; and with a start of 12 or 13 let the number increase until it reaches, as I think it can, at present 25 or 30 oophorectomies.

[We beg leave to remark that it was the *Courier Record* that "quoted" Dr. Wilkinson, and not this Journal. We merely extracted what the *Courier Record* had attributed to him. Our object was also, to call out reports.—Ed.]

